# MR-proADM and MR-proANP levels in patients with acute pulmonary embolism

**DOI:** 10.2478/jomb-2019-0049

**Published:** 2020-09-02

**Authors:** Önsel Öner, Figen Deveci, Selda Telo, Mutlu Kuluöztürk, Mehmet Balin

**Affiliations:** 1 Firat University, School of Medicine, Department of Pulmonary Medicine, Elazig, Turkey; 2 Firat University, School of Medicine, Department of Biochemistry, Elazig, Turkey; 3 Firat University, School of Medicine, Department of Cardiology, Elazig, Turkey

**Keywords:** pulmonary embolism, Mr-proADM, MRproANP, mortality, plućna embolija, Mr-proADM, MRproANP, smrtnost

## Abstract

**Background:**

The aim of this study was to determine levels of Mid-regional Pro-adrenomedullin (MR-proADM) and Mid-regional Pro-atrial Natriuretic Peptide (MR-proANP) in patients with acute pulmonary embolism (PE), the relationship between these parameters and the risk classification in addition to determining the relationship between 1and 3month mortality.

**Methods:**

82 PE patients and 50 healthy control subjects were included in the study. Blood samples for Mr-proANP and Mr-proADM were obtained from the subjects prior to the treatment. Risk stratification was determined according to sPESI (Simplified Pulmonary Embolism Severity Index). Following these initial measurements, cases with PE were assessed in terms of all causative and PE related mortalities.

**Results:**

The mean serum Mr-proANP and Mr-proADM levels in acute PE patients were found to be statistically higher compared to the control group (p < 0.001, p < 0.01; respectively) and statistically significantly higher in high-risk patients than low-risk patients (p < 0.01, p < 0.05; respectively). No statistical difference was determined in high-risk patients in case of sPESI compared to low-risk patients while hospital mortality rates were higher. It was determined that the hospital mortality rate in cases with Mr-proANP ≥ 123.30 pmol/L and the total 3-month mortality rate in cases with Mr-proADM ≥ 152.2 pg/mL showed a statistically significant increase.

**Conclusions:**

This study showed that Mr-proANP and MRproADM may be an important biochemical marker for determining high-risk cases and predicting the mortality in PE patients and we believe that these results should be supported by further and extensive studies.

## Abstract (sr)

**Uvod:**

Cilj ove studije bio je da utvrdi nivo midregionalnog proadrenomedulina (MR-proADM) i midregionalnog proatrijalnog natriuretskog peptida (MR-proANP) kod pacijenata sa akutnom plućnom embolijom (PE), odnos između ovih parametara i klasifikaciju rizika uz utvrđivanje odnosa smrtnosti od 1 do 3 meseca.

**Metode:**

U ispitivanje su uključena 82 pacijenta sa PE i 50 zdravih ispitanika. Uzorci krvi za Mr-proANP i Mr-proADM su uzeti od subjekata pre tretmana. Stratifikacija rizika određena je prema sPESI (pojednostavljeni indeks ozbiljnosti plućne embolije). Nakon ovih početnih merenja, slučajevi sa PE su ocenjivani u smislu svih uzroka i smrtnosti povezanih sa PE.

**Rezultati:**

Otkriveno je da su prosečni nivo Mr-proANP u serumu kod pacijenata sa akutnom PE statistički viši u poređenju sa kontrolnom grupom (p < 0,001, p < 0,01; respektivno) i statistički značajno veći kod visoko rizičnih pacijenata u poređenju sa pacijentima niskog rizika (p < 0,01, p < 0,05; respektivno). Nije utvrđena statistička razlika kod visoko rizičnih pacijenata u slučaju sPESI u poređenju sa pacijentima niskog rizika, dok je stopa bolničke smrtnosti bila veća. Utvrđeno je da stopa smrtnosti u bolnici u slučajevima sa Mr-proANP ≥ 123,30 pmol/L i ukupna tromesečna stopa smrtnosti u slučajevima sa Mr-proADM ≥ 152,2 pg/mL pokazuje statistički značajno povećanje.

**Zaključak:**

Ova studija je pokazala da Mr-proANP i MRproADM mogu biti važan biohemijski pokazatelj za određivanje slučajeva visokog rizika i predviđanja smrtnosti kod pacijenata sa PE i verujemo da bi ovi rezultati trebali biti podržani daljim i opsežnim studijama.

## Introduction

Classification of the Pulmonary embolism (PE) patients for early mortality as a high-risk, intermediate-risk or low-risk determines the treatment options and prognosis [1]. Clinical parameters [Pulmonary Embolism Severity Index (PESI), simplified PESI score (sPESI)], right ventricular dysfunction (RVD) markers, thrombotic load markers and myocardial damage markers in hemodynamically stable acute symptomatic PE cases are the most commonly used prognostic factors. In the determination of the risk of early poor prognosis in normotensive PE cases, none of the findings, such as high risk in clinical scoring, presence of RVD or increased levels of cardiac markers, are sufficient by itself. It is believed that prognostic evaluation results will be strengthened if these methods are used together [2].

Adrenomedullin (ADM) is a vasodilating peptide released from endothelial tissue in organs such as lungs, heart and gastrointestinal tract. It has natriuretic and antiproliferative effects. ADM is released in a pro-ADM form that is an inactive precursor. Midregional pro-ADM (MR-proADM), a more stable peptide, was found to be an important prognostic marker [3]. A-type natriuretic peptide (ANP) from the natriuretic peptide family increases in inflammatory conditions and hemodynamic stress. MR-proANP, the Midregional fragment of ANP, is more stable in blood than ANP. MR-proANP and MR-proADM were reported to have a significant diagnostic and prognostic value in a series of cases with acute dyspnea [4].

In our study, the aim was to determine the levels of the new cardiac markers, MR-proADM and MR-proANP, in acute PE cases. Additionally, it was aimed to determine the relationship between these parameters and the severity of disease in addition to risk classification and mortality from 1^st^ and 3^rd^ months in hospital and total 3-month mortality rate.

## Materials and Methods

### Patient population and study design

Eighty-two patients with acute PE cases who were referred to Chest Disease Polyclinic and Emergency Service in the Medicine Faculty of Firat University and diagnosed with PE by multidetector pulmonary CT angiography were included in the study, and 50 gender-and age-matched healthy sub-jects were included in the study as the control group. Ethical approval was obtained by the institutional review board (27.12.2016-178051). Significant heart valve disease, acute coronary syndrome or left ventricular ejection fraction less than 40% were excluded from the study. Demographic data and physical examination findings of PE cases were recorded. Blood samples for the MR-proADM and MR-proANP, and arterial blood gases (ABG) samples were obtained prior to the initiation of the treatment in PE patients and healthy controls. Furthermore, plasma D-dimer, troponin I (TnI) and serum brain natriuretic peptide (BNP) values, which were routinely obtained to determine the risk stratification in acute PE patients, were recorded. The severity of acute PE was determined by systemic systolic blood pressure, initial echocardiographic evaluation, RVD findings, plasma TnI and BNP levels as indicated in the European Society of Cardiology PE Guidelines [5]. The patients with a sPESI risk score of 0 were regarded as low-risk patients, and those with a sPESI score ≥ 1 were regarded as high-risk patients. After these initial measurements, acute PE patients were evaluated within a period of hospitalization for all the reasons and PE-related mortality on the 1^st^ and 3^rd^ months and total mortality rate.

### Patients' follow-up

All patients completed follow-up at 1^st^ and 3^rd^ months after enrollment. Their follow-up included one telephone interview and two face-to-face evaluations for observation during the 3 months of study participation. Then, semi-annual contacts, alternating between face-to-face evaluations (clinic visits or home visits for housebound patients) and telephone calls as well as periodic reviews of patients' hospital charts were conducted. During each visit/contact, the researchers included in the study interviewed patients and/or patients' relatives to obtain information about mortality.

### Arterial blood gas examination (ABG)

ABG samples collected from the radial artery in room temperature were examined by blood gas analysis device (Rapidlab 348, Biobak; Bayer Diagnostic, UK).

### Echocardiography (ECHO)

Pulmonary artery pressure (PAP) and RVD were evaluated by Doppler ECHO in patients with acute PE. ECHO was performed by a single 3.4 MHz transducer probe with two-dimensional, classical and tissue Doppler. sPAPs > 36 mmHg on the echocardiographic examination were regarded as pulmonary hypertension (PH) [6]. RVD was defined when at least one of the following conditions were present: right ventricle hypokinesia (asymmetrical or delayed contraction), systolic paradoxical movement in the septal wall, right ventricular dilation (end-diastolic diameter >30 mm, or right/left ventricle diameter ratio > 1) [7].

### Biochemical Analyses

Routine biochemical measurements were performed in our central laboratory (ADVIA 2400, Siemens Healthcare Diagnostics Inc., Tarrytown, USA). Serum BNP and TnI concentrations were performed on the ADVIA Centaur XP immunoassay analyzer (Siemens Healthcare Diagnostics Inc., Tarrytown, NY). Plasma D-dimer levels were assessed by using the BCS XP coagulation analyzer (Siemens Healthcare Diagnostics, Marburg, Germany).

Serum MR-proADM and MR-proANP levels were measured with a commercially available kit using an ELISA, Human MR-proADM ELISA kit (Catalogue No: 201-12-7275 Sunred Biological Technology Co. Ltd, Shanghai) with a low sensitivity limit of 2.839 pg/mL. The samples were measured in duplicates in a single experiment. The intra-and interassay coefficients of variance of this kit are < 10% and < 12%, respectively. The detection range of MR-proADM was 3-900 pg/mL, Human MR-proANP ELISA kit (Catalogue No: 201-12-6282 Sunred Biological Technology Co. Ltd, Shanghai with a low sensitivity limit of 1.863 pg/mL, and the detection range of MR-proANP was 2-400 pmol/L.

### Statistical analysis

In the statistical analysis process, IBM Statistical Product and Service Solutions version 21.0 (IBM SPSS Statistics 21 program, Armonk, NY, USA) software was used. Results were expressed as the mean ± standard deviation and percentage. A p-value of < 0.05 was deemed statistically significant. *X^2^* test was used to determine the gender difference. Statistical analysis was performed using Kruskal-Wallis test for multiple-group comparisons; Mann-Whitney U test was performed to test any observed differences for significance. Spearman's correlation was used to assess the nonparametric data. Receiver operating characteristic (ROC) curves were plotted to demonstrate the sensitivity and specificity of the evaluated MR-proADM and MR-proANP.

## Results

Eighty-two patients with acute PE (40 (48%) males and 42 (51.2%) females with a mean age of 64.96 ± 17.24) and 50 healthy controls (23 (46%) males and 27 (54%) females with a mean age of 66.16 ± 14.06) were included in the study. There was no significant difference in age (p > 0.05) and gender (p > 0.05, *X^2^* = 0.096) between the groups.

According to the sPESI index of patients with acute PE, 27 (20.30%) were at a low risk (Mean age of 56.19 ± 17.10 and the male / female ratio of 15 (55.6%)/12 (44.4%)) and 55 (41%) were at a high risk (Mean age of 69.27 ± 15.73 and the male/female ratio of 25 (45.5%)/30 (54.5%)). There was no significant difference in gender (p > 0.05, *X^2^* = 0.096) between high-and low-risk sPESI groups while the mean age of the high-risk cases was statistically higher than the low-risk cases (p < 0.01).

Of 82 patients with acute PE, 7 (8.53%) died during their stay in the hospital. Additionally, 3 of the patients (3.65%) died during the 1-month follow-up while another 3 (3.65%) died during the 3-month follow-up. All deaths were due to PE-related mortality (right heart failure, hemodynamic collapse, major bleeding, etc.). Hematological parameters of patients with acute PE were presented in Table 1.

**Table 1:** Hematological parameters of patients with acute pulmonary embolism ^a^ p < 0.01; when compared to low-risk acute PE patients, ^b^ p < 0.05; when compared to low-risk acute PE patients,^c^ p < 0.001: when compared to control group, ^d^ p < 0.01; when compared to control group.

	Total Acute PE (n = 82)	sPESI ≥ 1 High risk (n = 55)	sPESI < 1 Low risk (n = 27)	Control (n = 50)
D-dimer (mg/L)	4882.5 ± 5268.5	5617.6 ± 5871.7	3385.03 ± 3375.7	–
BNP (ng/mL)	4022.1 ± 6533.7	4854.6 ± 6297.3^a^	2326.2 ± 6795.4	–
TnI (ng/mL)	0.14 ± 0.46	0.19 ± 0.54^b^	0.06 ± 0.20	–
WBC (10^3^/mL)	8358.4 ± 4321.6	8887.4 ± 2817.2	7281.4 ± 2817.2	–
Plt (10^3^/mL)	268.1 ± 103.1	287.2 ± 110.7^b^	229.3 ±73.03	–
sPAP (mmHg)	42.2 ± 19.2	44.9 ± 20.08^b^	36.7 ± 16.3	–
PaO_2_ (mmHg)	67.1 ± 20.7	62.5 ± 20.3^a^	76.5 ± 18.7	–
PaCO_2_ (mmHg)	31.8 ± 5.9	31.6 ± 6.2	32.1 ± 5.2	–
pH	7.42 ± 0.06	7.43 ± 0.07	7.42 ± 0.04	–
SaO_2_ (%)	88.6 ± 10.2	86.5 ± 11.1^a^	92.89 ± 6.1	–
MR-proANP (pmol/L)	126.09 ± 38.1^c^	136.1 ± 38.4^a^	105.6 ± 28.5	79.6 ± 26.7
MR-proADM (pg/mL)	178.5 ± 82.2^d^	189.4 ± 87.8^b^	156.3 ± 65.4	142.1 ± 66.1

**Figure 1:**
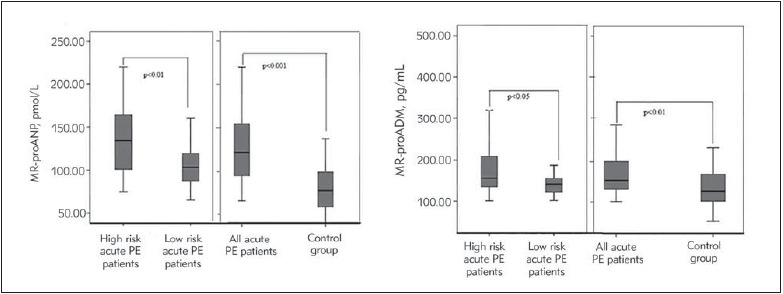
Mean serum MR-proANP and MR-proADM levels of cases

The mean serum MR-proANP and MR-proADM concentrations were statistically higher in patients with acute PE compared to the control group and significantly higher in patients with high clinical risk than those with low clinical risk (Figure 1, Table 1).

Significant differences were observed between sPESI ≥ 1 and sPESI < 1 groups regarding the BNP, TnI, platelet levels and sPAP (Table 1). There was no statistically significant difference in the hospital mortality between sPESI high-risk patients and low-risk patients while the hospital mortality rate was high in high-risk patients. Additionally, there was no statistically significant difference in 1^st^ -month, 3^rd^-month and 3-month total mortality rates between sPESI high-and low-risk groups (Table 2).

**Table 2:** The hospital, 1 month, 3 months and total 3-month mortality rates in high and low-risk groups according to sPESI

sPESI	Hospitalmortality n (%)		1^th^ month mortality n (%)		3^th^ month mortality n (%)		Total mortality n (%)	
	Ex	Live	Ex	Live	Ex	Live	Ex	Live
High risk (n = 55)	6(10.9)	49(89.1)	3(6.1)	46(93.9)	2(4.3)	44(95.7)	11(20)	44(80)
Low risk (n = 27)	1(3.7)	26(96.3)	0(0)	26(100)	1(3.8)	25(96.2)	2(7.4)	25(92.6)
*X^2^*, p value	1.204, > 0.05		1.658, > 0.05		0.010, > 0.05		2.153, > 0.05	

**Figure 2:**
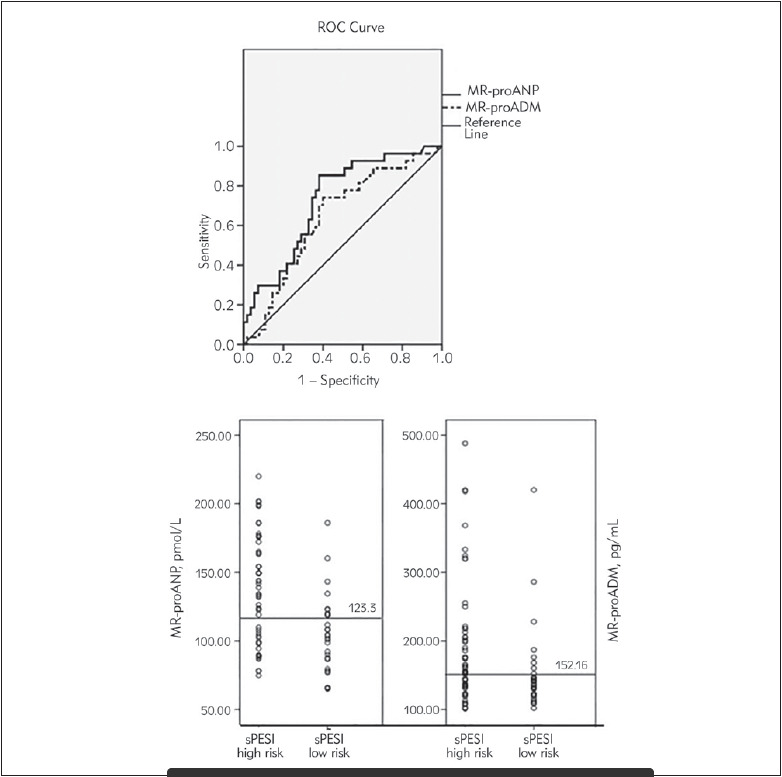
The cut-off levels of MR-proANP and MR-proADM in patients with high-low risk acute PE according to sPESI and receiver operating characteristic (ROC) curve indicating sensitivity and specificity of MR-proANP and MR-proADM to predict of high risk patients

When cut-off was taken as ≥ 123.3 pmol/L for MR-proANP with ROC analysis for predicting the high risk of sPESI in patients with acute PE, AUC was found to be 0.727 (95% CI; 0.615-0.89, p < 0.01). The sensitivity was 85% while the specificity was 62%. Furthermore, for the cut-off of ≥ 152.2 pg/mL in MR-proADM, AUC was 0.643 (95% CI;0. 518-0.767, p < 0. 01) and the sensitivity was 74% with a specificity of 60% (Figure 2).

After classifying the patients into two groups according to the cut off value as 123.3 pmol/L for MR-proANP, the hospital mortality rate was found to be statistically higher in patients with MR-proANP ≥ 123.3 (Table 3). Additionally, the total mortality rate was found to be statistically higher in patients with MR-proADM ≥ 152.2 pg/mL (Table 4).

**Table 3:** Hospital mortality rate was found to be statistically higher in patients with MR-proANP ≥ 123.3 pmol/L

MR-proANP	Hospitalmortality n (%)		1^th^ month mortality n (%)		3^th^ month mortality n (%)		Total mortality n (%)	
	Ex	Live	Ex	Live	Ex	Live	Ex	Live
< 123.3 pmol/L (n=42)	1(2.4)	41(97.6)	1(2.4)	40(97.6)	2(5)	38(95)	4(9.5)	38(90.5)
≥123.3 pmol/L (n = 40)	6(15)	34(85)	2(5.9)	32(94.1)	1(3.1)	31(96.9)	9(22.5)	31(77.5)
*X^2^*, p value	** 4.178, < 0.05 **		0.574, > 0.05		0.157, > 0.05		2.586, > 0.05	

**Table 4:** Total 3-month mortality rate was found to be statistically higher in patients with MR-proADM ≥ 152.2 pg/mL

MR -proADM	Hospitalmortality n (%)		1^th^ month mortality n (%)		3^th^ month mortality n (%)		Total mortality n (%)	
	Ex	Live	Ex	Live	Ex	Live	Ex	Live
< 152.2 pg/mL (n = 40)	1(2.5)	39(97.5)	1(2.6)	38(97.4)	0(0)	38(100)	2(5)	38(95)
≥152.2 pg/mL (n= 42)	6(14.3)	36(85.7)	2(5.6)	34(94.4)	3(8.8)	31(91.2)	11(26.2)	31(73.8)
*X^2^*, p value	3.645, > 0.05		0.436, > 0.05		3.499, > 0.05		** 6.896, < 0.05 **	

When the correlation between MR-proANP and cardiac markers, and sPAP in patients with acute PE were evaluated; only a positive correlation was determined between MR-proANP and D-dimer levels (r = 0.234, p < 0.05). No correlation was determined between MR-proADM and any of the parameters.

When evaluated by logistic regression analysis, only MR-proADM value of ≥ 152.2 pg/mL was found to be an independent risk factor for 3-month total mortality (p < 0.05, OR: 6.742, (% 95 CI: 1. 389-32.717)).

## Discussion

Our study results showed that serum MR-proANP and MR-proADM levels were significantly higher in acute PE patients than in the control group and both of them was especially found to be statistically higher in sPESI ≥ 1 group. MR-proANP and MR-proADM values had a moderate specificity and sensitivity in predicting high sPESI score. Additionally, increased MR-proADM values were an independent risk factor for 3-month total mortality rate in acute PE patients.

The prognostic value and the role of MR-proADM in risk stratification were demonstrated in patients with acute heart failure [8] [4]. Furthermore, recent observational studies showed that proADM is a powerful independent prognostic factor in long-term non-survival in COPD patients [9] [10] [11] [12]. In the literature, several studies evaluated the role of pro-ADM in patients with community-acquired pneumonia (CAP) [13]. According to the study results, MR-proADM was found as a good predictor of short-and long-term allcause mortality in CAP patients. A recently published prospective multicenter study demonstrated that MR-proANP and MR-proADM were associated with exercise variables and prognosis in pulmonary arterial hypertension (PAH) and chronic thromboembolic pulmonary hypertension (CTEPH) [14]. MR-proADM is a good prognostic indicator of mortality, and it may have a contributory effect in the risk stratification of acute dyspnea patients. Previous studies also reported similar AUC values (0.81) for MR-proADM in predicting 30-day mortality in acute dyspnea patients [15] [16]. Another study, which evaluated the diagnostic and prognostic value of MR-proADM and MR-proANP in patients with acute dyspnea, found that MR-proANP had a diagnostic value in patients with acute dyspnea while both MR-proANP and MR-proADM were independent prognostic factors for 4 years follow-up [17]. The study of Heining et al. [18] demonstrated that MR-proADM was the best predictor of non-survival patients with acute heart failure while pneumonia and values ≥1.5 nmol/L were associated with a high risk of death. On the other hand, MR-proANP was found as predictors for the diagnosis of PE in this study, while its predictive potential could not be identified due to the low incidence of this final diagnosis. The predictive value of natriuretic hormones was reported for PE, and its severity and outcome [19] [20]. Increased levels of the natriuretic peptides, such as ANP, BNP and N-ANP, were found in patients with a high probability of PE on radionuclide scanning [19]. A progressive incremental rise in natriuretic peptide levels with increasing degrees of ventilation-perfusion mismatch within the lung was reported. Therefore, it was believed that there was a direct relationship between perfusion defects and release of natriuretic peptides [19]. Few previous studies examined the prognostic value of MR-proADM in acute PE patients. These studies reported that MR-proADM was an independent predictor of PE-related mortality [21] [22]. It was demonstrated that the prognostic accuracy of MRproADM tended to increase over time, C-statistics reaching to 0.84 for PE-related mortality at 24 months while being a nonsignificant predictor for 1 month [22]. Moreover, the prognostic superiority of MR-proADM over NT-proBNP was also described for acute PE and MR-proADM levels were found significantly higher in high-risk acute PE patient compared to hemodynamically stable patients. Therefore, it was thought that plasma levels of MR-proADM reflect the severity ofacute PE [21]. In our study, MR-proADM value of ≥152.2 pg/mL was determined to be an independent risk factor for 3-month total mortality through logistic regression analysis. Similar to the study of Pedowska-Włoszek et al. [21] we found that MR-proADM levels were elevated in acute PE patients compared with the control group. Additionally, when we evaluated the role of new cardiac markers, MR-proADM and MR-proANP, for risk stratification in patients with acute PE, we observed that both markers had an acceptable AUC value. We demonstrated that increased MR-proANP might be related to hospital mortality. For this reason, we believe that MR-proADM and MR-proANP may be potential biomarkers in providing additional information on the risk stratification of patients with PE.

MR-proADM is a pluripotent regulatory vasoactive peptide related to a variety of cardiovascular, renal, metabolic, and immunomodulatory effects. It acts as a vasodilator, natriuretic, diuretic, antioxidative, anti-inflammatory, antimicrobial, and metabolic agent [12] [23]. Up-regulation of MR-proADM was also linked to hypoxia and inflammation [24]. Increased ADM levels in final-stage pulmonary disease may be related to the reflection of "high demand" for these compensatory/counter-regulatory effects [12] [25] [26] [27]. The possible physiopathological association between elevated levels of MR-proADM and mortality in PE may be explained by the cardiomyocytes stretch and hypoxia in promoting ADM gene expression [28] [29]. Weak positive correlations with mean pulmonary arterial pressure and ADM levels were found, and this indicated to the role of severity of vascular impairment at increased ADM levels [27].

### Study limitations

Our study group consisted of a relatively small group in a single centre. Furthermore, the study group did not include patients with significant heart valve disease, acute coronary syndrome or left ventricular ejection fraction less than 40%. For this reason, our study results should be interpreted carefully. Furthermore, MR-proADM and MR-proANP levels were only measured initially. We could not perform follow-up measurements of the biomarkers. Therefore, we could not make any comments on the marker kinetics and monitoring of treatment response. Our study covered a short follow-up period. Long-term follow-up of these markers may provide more accurate information about their prognostic value in patients with PE.

## Conclusions

The findings of our study indicated that MR-proANP and MR-proADM could be a promising new biomarker for determining high-risk cases and predicting the short-term mortality of PE patients. Integration of these markers into sPESI scores may be a contributing element in the determination of the severity of PE in hemodynamically stable patients. Further research is necessary to determine whether MR-proANP and MR-proADM can be used with other validated markers.

## Conflict of interest statement

The authors stated that they have no conflicts of interest regarding the publication of this article.
